# Potential application mechanism of traditional Chinese medicine in treating immune checkpoint inhibitor-induced colitis

**DOI:** 10.3389/fimmu.2024.1366489

**Published:** 2024-04-10

**Authors:** Jing Wang, Ziyue Guo, Mengyi Shen, Qi Xie, Hongjie Xiang

**Affiliations:** ^1^ College of Traditional Chinese Medicine, Shandong Second Medical University, Weifang, China; ^2^ Department of Traditional Chinese Medicine, The First Affiliated Hospital of Shandong First Medical University & Shandong Provincial Qianfoshan Hospital, Jinan, China; ^3^ Shangdong First Medical University & Shangdong Academy of Medical Sciences, Jinan, China; ^4^ Department of Oncology, The First Affiliated Hospital of Shandong First Medical University & Shandong Provincial Qianfoshan Hospital, Shandong Key Laboratory of Rheumatic Disease and Translational Medicine, Shandong Lung Cancer Institute, Jinan, China

**Keywords:** traditional Chinese medicine, immune checkpoint inhibitor-induced colitis, potential application, mechanisms, immune

## Abstract

Cancer ranks among the foremost causes of mortality worldwide, posing a significant threat to human lives. The advent of tumor immunotherapy has substantially transformed the therapeutic landscape for numerous advanced malignancies, notably non-small cell lung cancer and melanoma. However, as immune checkpoint inhibitors (ICIs) are increasingly applied in clinical settings, a spectrum of undesired reactions, termed immune-related adverse events (irAEs), has emerged. These adverse reactions are associated with immunotherapy and can result in varying degrees of harm to the human body. Among these reactions, Immune checkpoint inhibitor-induced colitis (ICIIC) stands out as one of the most prevalent clinical adverse events. In contemporary times, traditional Chinese medicine (TCM) has demonstrated remarkable efficacy in addressing various maladies. Consequently, investigating the potential application and mechanisms of Chinese medicine in countering immune checkpoint inhibitor-induced colitis assumes significant importance in the treatment of this condition.

## 1 Introduction

In recent years, the global cancer incidence has been steadily rising. Traditional cancer treatments include surgery, radiotherapy, chemotherapy, targeted therapy, and interventional therapy (1). Immunotherapy has become the fifth pillar of cancer management alongside surgery, chemotherapy, radiotherapy, and targeted therapy (2). Immune checkpoint inhibitors (ICIs) include nivolumab, pembrolizumab (PD-1 monoclonal antibodies), atezolizumab, avelumab, durvalumab (PD-L1 monoclonal antibodies), and ipilimumab (CTLA-4 inhibitor), among others (3). The side effects of immune checkpoint inhibitors have gained attention, affecting various systems like skin, gastrointestinal tract, heart, and more. Gastrointestinal immune-related adverse events (GI-irAEs)is a common issue (4). Traditional Chinese medicine (TCM), with a 2,000-year history, complements conventional cancer treatments effectively. TCM combined with antitumor therapy suppresses tumors, reduces drug resistance, and improves patient quality of life (1). Chinese herbal medicine is affordable, accessible, and minimally disruptive, making it widely accepted by patients. Investigating TCM’s potential in addressing immune checkpoint inhibitor-induced colitis offers new treatment approaches, enhancing patient quality of life and clinical care for this condition.

## 2 Status quo of ICIIC

The primary symptoms of immune checkpoint inhibitor-induced colitis (ICIIC) include watery stool and non-bloody diarrhea, with abdominal pain, blood in the stool, nausea, vomiting, weight loss, and fever (1). Studies indicate that patients in the αCTLA-4 group have a higher incidence of GI-irAEs and more frequent diarrhea than patients in the αPD-1/PD-L1 group. Wang et al. reported colitis incidences of 9.1% with αCTLA-4 monotherapy, 1.3% with anti-PD-1/PD-L1 monotherapy, and 13.6% with combination therapy (2).

In a previous study (4), it was found that GI-irAEs typically occurred around 6-7 weeks after starting treatment with ipilimumab, an anti-CTLA-4 antibody. Another study showed that colitis began at 25.4 weeks in patients treated with anti-PD-1 monotherapy, compared with 7.2 weeks in patients treated with combination therapy (5). The incidence of diarrhea and colitis in patients treated with ipilimumab is approximately 30%-40% (6). Colitis typically affects the descending colon, but in certain instances, enteritis without colonic involvement occur, might leading to small bowel obstruction (7).

In most cases, mild GI-irAEs can resolve on their own or with close monitoring, and may not require discontinuation of ICIs. However, moderate to severe symptoms can lead to significant morbidity, affecting the patient’s nutritional and fluid balance status, and potentially necessitating hospitalization. This can also impact the patient’s eligibility for receiving further treatment (8). GI-irAEs can be classified into four grades of increasing severity in clinical practice, with corresponding diagnostic examinations and management strategies. Grades 1 and 2 involve mild to moderate diarrhea, while grades 3 and 4 are considered severe. Severe diarrhea can be accompanied by symptoms such as abdominal pain, rectal bleeding, and mucus in stool (9). At this critical stage, it can become life-threatening and potentially result in bowel perforation (7). Diagnostic evaluation for GI-irAEs includes stool microbiological culture and sensitivity testing, as well as colonoscopy if colitis is suspected or if diarrhea persists despite corticosteroid therapy (9). Endoscopy typically reveals inflammatory changes in the gastrointestinal tract, including erythema, inflammatory exudates, granularity, loss of vascularity, and ulcerations. Management usually involves the use of corticosteroids. In cases of steroid-refractory grades 3 and 4 diarrhea, second-line immunosuppressive therapies like budesonide, vedolizumab, or aminosalicylates have been reported as successful and safe options. Fecal microbiota transplantation (FMT) has also been used as a third-line therapy in some cases (10).

## 3 The mechanism of ICIIC

The exact pathogenic mechanism of systemic adverse reactions caused by ICIs is not fully understood, but it is believed that over-activated T cells contribute to the development of irAEs. The inhibition of CTLA-4 and PD-1 pathways, which act as negative regulators of T cell activation, can lead to irAEs. CTLA-4 functions to inhibit T cells at the initial stage of activation in the lymph nodes, whereas PD-1 regulates previously activated T cells in peripheral tissues or at tumor sites (11). The interaction of CTLA-4 on naive T cells to B7 on antigen-presenting cells (APCs) triggers an inhibitory signal upon T cell activation. Additionally, CTLA-4 can prompt transendocytosis of B7 molecules on APCs, further inhibiting T cell activation (12). PD-1 and its ligand PD-L1 interaction also leads to T cell inactivation. There may be a difference in the risk of colitis between PD-1/L1 inhibitors and CTLA-4 inhibitors, suggesting that CTLA-4 more thoroughly inhibits T cell activation (13). However, it is important to note that irAEs involve a complex interplay between various components of the immune system, including T cells, humoral immunity, and autoimmune diseases. Emerging evidence also suggests a possible association between the composition of the intestinal microbiota and the efficacy of immune checkpoint blockade (11, 14). The intricate relationship between the composition and ecology of the gut microbiota and the activation of specific T cells has been elucidated in prior research (15). Staphylococcal enterotoxin B has been demonstrated to elicit T cells activation, subsequently triggering the production of IFN-γ and IL-2 by these cells (16). A study has shown that human gut *Actinobacterium Eggerthella lenta* can induce intestinal Th17 activation by unblocking the Th17 transcription factor, exacerbating colitis in mice (17). In the therapeutic management of colitis induced by dextran sodium sulfate (DSS), FMT has shown promise in selectively reducing the representation of CD4^+^ and CD8^+^ T cells in the colon, thereby supporting intestinal homeostasis. This suggests a clear association between the gut microbiome and the modulation of T cell activity (18). Higher relative abundance of the Bacteroidetes phylum in the gut microbiota has been linked to a reduced rate of ipilimumab-induced colitis (19). FMT has been reported as a successful treatment for severe colitis associated with ICIs (20). These findings highlight the importance of regulating T cell activation, the intestinal microenvironment, and the gut microbiota in the management of irAEs.

## 4 The current treatment for ICIIC

The current clinical treatment for GI-irAEs mainly involves the use of steroid drugs. In refractory cases, infliximab and vedolizumab are commonly used. It has been observed that CTLA-4 inhibitors may require higher steroid doses compared to PD-1/PD-L1 inhibitors for the treatment of GI-irAEs (21). However, these drugs may not always be effective and can present challenges for physicians. The onset of GI-irAEs can vary widely, making it difficult to predict its development. The pathological features of GI-irAEs resemble those of chronic gastrointestinal inflammation, such as inflammatory bowel disease (IBD), ulcerative colitis (UC), and Crohn’s disease (CD) (22). ICIIC and IBD exhibit similarities in terms of endoscopy findings, histological evaluation, intestinal microbiota, and therapeutic approaches. However, the etiology of IBD is multifactorial, typically involving the intricate interplay of genetics, environmental factors, microbial factors, and immune-inflammatory responses. This complex interaction gives rise to a wide range of overlapping phenotypes, with each disease process displaying distinct clinical presentations (22). On the other hand, ICIIC commonly arises as a consequence of treatment with ICIs, and immunohistochemical analysis has revealed an elevated proportion of CD4^+^ cells and CD8^+^ cells in ICIIC (23, 24). It is crucial for physicians to quickly recognize and differentiate these conditions when managing immunosuppressive colitis to prevent worsening of the patient’s condition.

## 5 Potential treatment of TCM for ICIIC

**Figure 1:**
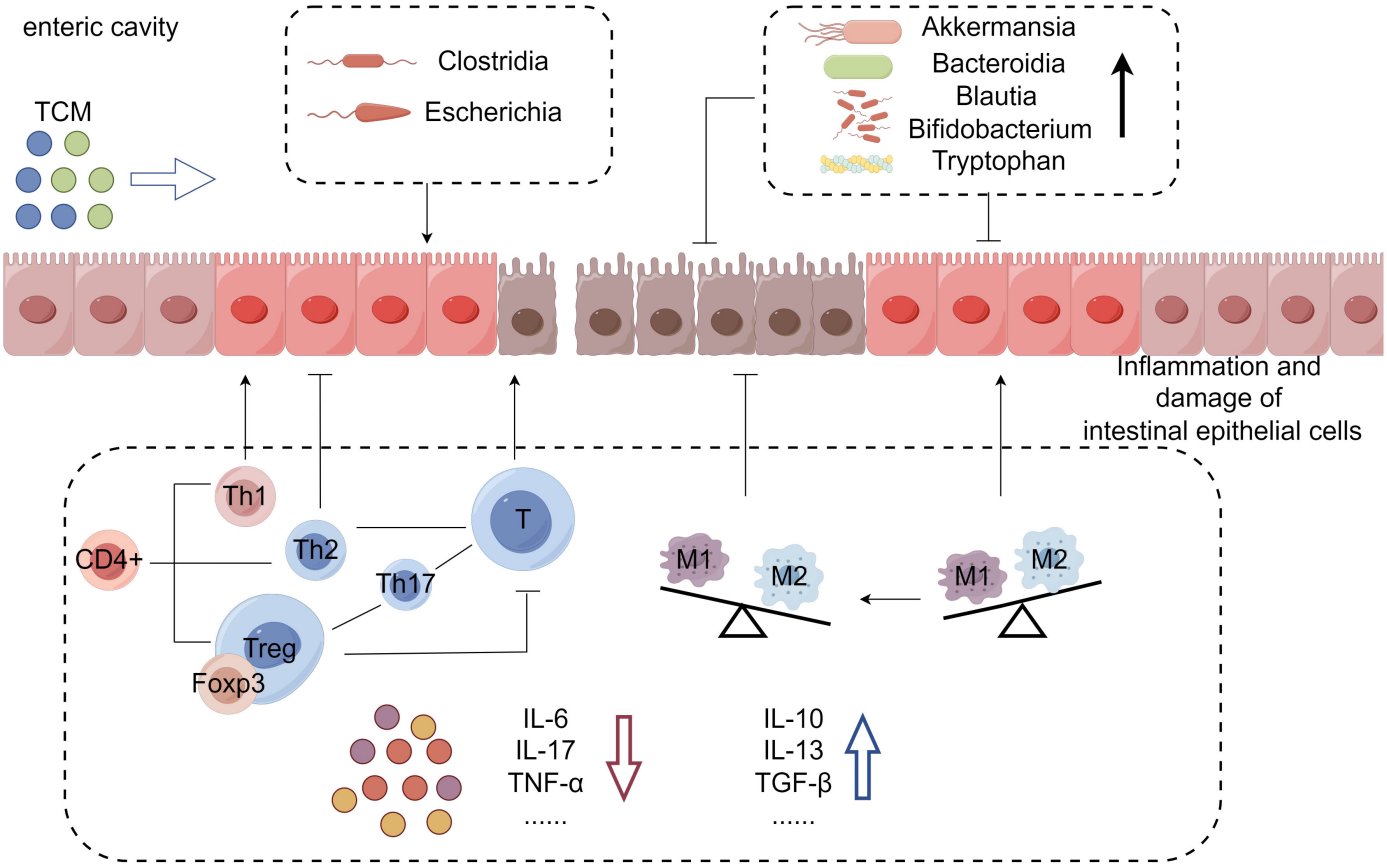
Mechanism of TCM in the treatment of immune checkpoint inhibitor-induced colitis: In this condition, overactivated T cells lead to intestinal epithelial inflammation, mucosal damage, barrier breakdown, and an imbalance of inflammatory factors. TCM can alleviate colitis symptoms by increasing anti-inflammatory factors, reducing pro-inflammatory factors, and altering intestinal flora and metabolites. This mechanism illustrates how TCM can effectively treat immune checkpoint inhibitor-induced colitis.

TCM has been used for centuries in China and East Asia and shows great potential in treating various complicated disease (25). Currently, there is limited research on the use of traditional Chinese medicine for the treatment of ICIIC. However, given the similarities between ICIIC and IBD in terms of endoscopic lesions, histopathological features, and treatment approaches (22), combined with TCM’s efficacy in treating IBD, it is plausible to consider the potential application of TCM in the treatment of ICIIC. Since the pathogenesis of ICIIC remains unclear, existing literature suggests that it may be associated with gastrointestinal immune inflammation and alterations in intestinal flora (20, 26–28). TCM can potentially target immune cells, the microenvironment, and intestinal flora effective treatment of ICIIC (**Figure 1** and **Table 1**). The regulation of immune cells by TCM may occur through the following avenues: a) Reactivation of regulatory T cells (Tregs) to mediate immunosuppression. b) Inhibition of hyperactive macrophages. c) Restoration of the balance between T helper cells (Th1/Th2). d) Other mechanisms (66).

**Table 1:** Potential mechanism of Chinese medicine in treating immune checkpoint inhibitor-induced colitis.

Name	Cell Type/Model	Mechanism of Effect	Effect	Refs
**Hyperoside (HYP)**	Dextran sulfate sodium-induced ulcerative colitis in mice		Th17↓, Treg↑, Th17/Treg balance	(29)
**Baicalin**	Trinitrobenzene sulphonic acid-induced UC rats model		Reduce Th17/Treg ratio	(30)
**Sophora flavescens Aiton, Kurarinone**	DSS-induced UC mouse model	Down-regulated JAK2/STAT3 signaling pathway	TNF-α, IL-6, IL-17A↓, IL-10, TGF-β1↑, regulate Treg/Th17	(31)
**Compound sophorae decoction**	DSS-induced UC mouse model		Treg, IL-10, Foxp3, TGF-β1↑, IL-17, IL-1β, IL-17A, IL-6↓; regulate Th17/Treg balance	(32)
**Kuijieling decoction**			Treg, Foxp3↑, IL-6R, IL-23R, Th17↓; regulate Th17/Treg balance	(33)
**Qingre Zaoshi Liangxue decoction**	TNBS-induced UC		IL-6, IL-17↓, Treg↑	(34)
**Tetrandrine (TET)**			Th1, Th2, Th17↓, iTreg↑	(35)
**Lactobacillus plantarum**	DSS-induced UC mouse model		TNF-α, IL-6, IL-17, IL-1β↓ IL-10, TGF-β1↑, regulation of Th1/Th2/Treg/Th17	(36)
**Paeoniflorin**		Inhibit DC function	IL-10, TGF-β↑, IL-17↓	(37)
**Banxia Xiexin** **decoction**	TNBS-induced colitis in rats		IL-4, IL-10, ↑ IL-1β, TNF-α, IL-17↓	(38)
**Luteolin**		Inhibition of p-STAT3 and activation of p-STAT6	IL-1β, IL-6, TNF-α↓, IL-10, IL-13↑; reduce M1 polarization and increase M2 polarization	(39)
**Baitouweng** **decoction**	DSS-induced colitis in mice	Suppress the activation of the IL-6/STAT3 signaling pathway	IL-1β, IL-6, TNF-α↓; inhibit M1 macrophage polarization	(40)
**Tiliroside**	DSS-induced colitis in mice		IL-1β, TNF-α↓; promote M2 macrophage polarization and inhibit M1 macrophage polarization	(41)
**Platycodin D**	DSS-induced colitis in mice		IL-10↑, TNF-α, IL-6, IL-1β↓; inhibit M1 polarization and promote M2 polarization	(42)
**Loganin**	DSS-induced colitis in mice		IL-6, TNF-α, and IL-1β mRNA, M1↓	(43)
**Tongxie-Yaofang formula**	DSS-induced colitis in mice	Inhibit the activation of NF-κB/NLRP3 signaling pathway	Reduce M1 polarization, and increase M2 polarization	(44)
**Dioscin**	DSS-induced colitis in mice		Reduce M1 polarization, and promote M2 polarization	(45)
**Huanglian-Houpo Decoction (HLHP)**			Regulate the polarization of macrophages to M2	(46)
**Cayratia japonica (CJ)**			Inhibit M1 polarization; promote M2-like polarization	(47)
**Berberine (BBR)**	DSS-induced colitis mice	Activate IL-4-STAT6 signaling pathway; inhibit TLR4-MyD88-NF-κB signaling pathway	Reduce M1; increase M2 polarization; restore the dysbacteria to normal level	(48)
**Didymin**	DSS-induced colitis in mice		Macrophage polarization from M1 to M2	(49)
**Wumeiwan (WMW)**	DSS-induced colitis in mice	Inhibit the activation of p38MAPK, NF-κB and STAT6 signaling pathways	Reduce M1 polarization; increase M2 polarization	(50)
**Cinobufacini**	DSS-induced colitis in mice		Reduce M1 numbers; increase M2 numbers; inhibit M1 polarization	(51)
**Methyl gallate (MG)**	DSS-induced colitis in mice	Inhibit TLR4/NF-κB signaling pathway	Prompt the polarization of macrophages from M1 to M2	(52)
**Buckwheat** **(F. esculentum) bee pollen extract (FBPE)**	DSS-induced colitis in mice		IL-10↑; reverse Th1/Th2 balance	(53)
**Platycodon grandiflorus polysaccharide (PGP)**	DSS-induced colitis in mice		Regulation of Th1/Th2 and Th17/Treg imbalance, IL-1β, TNF-α↓ IL-10↑	(54)
**Periplaneta americana (P. americana)**	DSS-induced colitis in mice	Inhibit TLR4/MAPK/NF-κB signaling pathway	IFN-γ, IL-2, TNF-α↓, IL-10↑; recover Th1/Th2 ratio	(55)
**Ginsenoside Rg1 (Rg1)**	DSS-induced colitis in mice		Increase the levels of tryptophan metabolites	(56)
**Rhine**			Up-regulate *Bacteroidetes*, *Lactobacillus*, IL-1β, IL-6, TNF-α↓	(57, 58)
**Oxyberberine (OBB)**	DSS-induced UC mice model	Inhibit TLR4-MyD88-NF-κB signaling pathway	Increase the abundance of *Bacteroides*; IL-6, IL-1β, IL-17, TNF-α, IFN-γ↓	(59)
**Huanglian and Houpo**	2,4,6-trinitrobenzene sulfonic acid-induced UC rats		Increase the abundance of probiotics such as *Akkermansia* and *Blautia*; increase *Allobaculum* and *Alloprevotella* richness	(60)
**Gegen-Qinlian decoction (GQD)**			Increase the abundance of *Akkermansia*, Bacteroides; enhance the diversity of gut bacterial species, TNF-α, IL-6, IL-1β, IL-4↓	(61, 62)
**Geng**	DSS-induced colitis in mice		Increase the abundance of beneficial probiotics such as *Bifidobacterium*, *Bacteroides*, and *Akkermansia*	(63)
**Pingkui enema**	TNBS-induced UC		Increasing *Bifidobacterium*, IL-8, TNF-α↓ IL-13↑	(64)
**Rabdosia serra** **(R. serra)**	DSS-induced colitis in mice		Increased the abundance of *Bacteroides*, *Lactobacillus*, restored the balance of Treg/Th17 to treat colitis	(65)

"↑" indicates an increase in the previously mentioned inflammatory factors, and "↓" indicates a decrease in the previously mentioned inflammatory factors.

### 5.1 Reactivation of regulatory T cells

Tregs, a subset of CD4^+^ T cells, play a crucial role in regulating autoimmune responses and maintaining immune balance. When activated, Tregs actively suppress the immune function of T lymphocytes. Foxp3 serves as a specific marker for Tregs and is essential in their development, peripheral expression, and functional maintenance, often referred to as the “primary regulator” of Tregs (67). In normal human biology, intestinal Tregs are essential for preserving immune stability in the gastrointestinal region by inhibiting abnormal immune reactions (68). Th17 cells, a type of T helper cells, are strongly linked to inflammation in the gut (66). Colitis typically presents with ulcer formation, infiltration by inflammatory cells, and an upregulation in pro-inflammatory cytokines such as IL-6, IL-12, IL-23. These pro-inflammatory factors contribute to intestinal inflammation and induce the conversion of naive T cells in the small intestine into Th17 cells, which release inflammatory cytokines like IL-17A and IL-17F. Notably, with the influence of TGF-β, intestinal DC can promote the induction of Tregs (68). Hyperoside (HYP) is found in a variety of plants such as Ligustrum phillideni, Hypericum, parsnips, and pecan. It has been shown to facilitate the differentiation of Tregs, suppress Th17 cells in cases of colitis, restore balance to the Th17/Treg axis, and ameliorate colitis (29). Baicalin, derived from Huangqin, a widely used Chinese herb in the treatment of ulcerative colitis, has been demonstrated to reduce the Th17/Treg ratio in TNBS-induced colitis, indicating its potential to alleviate colorectal inflammation (30). Triptolide (TPT), derived from the Chinese herb Tripterygium wilfordii, possesses strong immunosuppressive and anti-inflammatory characteristics. It efficiently inhibits the maturation of DCs. Additionally, TPT can increase the expression of DC-SIGN, promote the production of IL-10, and encourage DCs to adopt a tolerance-inducing phenotype. Consequently, TPT-treated DCs contribute to the expansion of Tregs (69). Various flavonoid compounds in the extract of Sophora flavescens (SFE) suppressed the productions of IL-6 and TNF-α in RAW 264.7 cells, with Kurarinone demonstrating significant potency. By downregulating the JAK2/STAT3 signaling pathway and controlling the differentiation of Th17 and Tregs, Kurarinone contributed to reestablishing the intestinal immune system’s balance in UC (31). The compound sophorae decoction, a Chinese herbal formula comprising six traditional Chinese herbs, demonstrated effective mitigation of colonic mucosal damage and reduction of IL-17A levels in colon tissue. In colitis-afflicted mice, it facilitated the development of Tregs, thereby modulating the Th17/Treg balance. The decoction upregulated the expression of IL-10, downregulated the concentration of IL-6, and increased TGF-β1 levels in colonic tissues, ultimately resulting in reduced colon inflammation in mice (32). The Kuijieling decoction, a TCM known for its impressive effectiveness in treating ulcerative colitis, functions by increasing the expression of Smad3 and Foxp3 to support Tregs differentiation. At the same time, it suppresses IL-6R and IL-23R genes expressions and the production of RORγt, resulting in the inhibition of Th17 differentiation and ultimately lowering the Th17/Treg cell ratio (33). The Qingre Zaoshi Liangxue decoction (QrLx), a Chinese herbal formula frequently employed for treating UC, has undergone extensive research. Studies have demonstrated that QrLx efficiently suppresses the expression of the inflammatory cytokine IL-6, as well as STAT3 and RORγt, which are associated with pro-inflammatory responses. Conversely, QrLx enhances the expression of Foxp3, a critical regulator of Tregs. These actions contribute to a considerable reduction in the ratio of Th17 cells and an increase in the ratio of Tregs (34). Tetrandrine (TET) is an anti-inflammatory bisbenzylisoquinoline alkaloid derived from Stephania tetrandra S. Moore, utilized in the management of autoimmune diseases, hypertension, and silicosis. In an experimental study, TET was found to strongly suppress the differentiation of pro-inflammatory Th1, Th2, and Th17 cells *in vitro* and *in vivo*, while leaving the differentiation of immunosuppressive iTreg cells unaffected (35). Lactobacillus plantarum was utilized to ferment Astragalus, a traditional Chinese medicine, and its potential therapeutic impact on DSS-induced colitis in mice was explored. The research uncovered that the fermented Astragalus (FA) reduced pro-inflammatory factors like TNF-α, IL-1β, IL-6, and IL-17, while enhancing anti-inflammatory factors like IL-10 and TGF-β. These observations imply that FA can adjust the inflammatory status by controlling the balance of cytokines associated with Th1, Th2, Th17, and Treg immune responses (36). In animal studies, paeoniflorin, derived from white paeony root, has demonstrated effectiveness in treating diseases resulting from immune overreactions by suppressing immune activation and response (70). A recent study revealed that paeoniflorin inhibits the maturation and immunostimulatory function of mouse bone marrow-derived DCs by enhancing the secretion of IL-10 and TGF-β, and decreasing IL-12 release and co-stimulatory molecules expression (37). Banxia Xiexin decoction has been shown to possess anti-inflammatory and antioxidant properties (71). The study indicated that the administration of Banxia Xiexin decoction decreased the disease activity index (DAI) score and colonic mass index, thereby alleviating histopathological damage in colitis-afflicted mice. Furthermore, it elevated the levels of anti-inflammatory factors such as IL-4 and IL-10, while decreasing the levels of pro-inflammatory factors such as IL-1β, TNF-α and IL-17 (38).

### 5.2 Inhibition of hyperactive macrophages

Macrophages are a multifaceted group of cells capable of performing a wide range of functions. In the intestines, macrophages play a vital role in maintaining immune balance and regulating inflammation. Overactive macrophages are involved in the body’s inflammatory response. Macrophages can be categorized into two phenotypes: M1 and M2. M1 macrophages primarily contribute to the initiation of inflammation and tissue damage, while M2 macrophages protect tissues from inflammation by regulating immune responses (72). Stimulation with lipopolysaccharide (LPS) and/or IFN-γ polarizes macrophages into M1 phenotype. These M1 macrophages produce inducible nitric oxide synthase (iNOS), IL-1, IL-6, and TNF-α. They also highly express CD86 and other proinflammatory cytokines, which aid in microbial clearance. On the other hand, stimulation with IL-4 or IL-13 polarizes macrophages into M2 phenotype. These M2 macrophages secrete anti-inflammatory factors such as arginase 1 (Arg-1) and IL-10. Additionally, they show increased expression of scavenger receptor CD163 or CD206, which helps alleviate inflammation and promote tissue regeneration. It is worth noting that macrophage polarization is regulated by various signaling pathways (39). Reducing the number of macrophages in the body or converting M1 macrophages to M2 macrophages might effectively alleviate the inflammatory response in ICIIC.

Luteolin, a natural flavonoid compound found in vegetables, fruits, and herbs, exhibits strong anti-inflammatory effects and possesses various beneficial biological properties, including anti-tumor, antioxidant, anti-infective, immunomodulatory, and cardioprotective activities. Studies have shown that luteolin not only reduces M1 polarization of macrophages and increases M2 polarization of macrophages, but also reduces pro-inflammatory IL-6 and TNF-α, and increases anti-inflammatory IL-10, thereby alleviating intestinal immune inflammation (39). In a study, it was demonstrated that Baitouweng decoction improved colon shortening, reversed body weight loss, and decreased DAI scores. Additionally, it was found to lower the levels of pro-inflammatory cytokines such as IL-1β, IL-6, and TNF-α. This decoction also ameliorated colonic pathological damage and inhibited M1 macrophage polarization. These effects were mediated by suppressing the activation of the IL-6/STAT3 signaling pathway in a DSS-induced colitis model (40). Tiliroside has been proven to promote M2 macrophage polarization while inhibiting M1 macrophage polarization. It achieves this by regulating macrophage polarization through the blocking of the glycolysis pathway. As a result, tiliroside possesses the ability to ameliorate pathological changes in the colon, increase survival rates, decrease DAI scores, and promote longer colon length (41). Platycodin D inhibits M1 macrophage polarization and promotes M2 macrophage polarization. It also protects intestinal barrier function and improves intestinal inflammation. Studies have shown that platycodin D is capable of suppressing peripheral and colonic tissue inflammation, reducing levels of IL-1β, IL-6, IL-10, and TNF-α, and alleviating colonic pathological damage (42). Loganin, an extract of Cornus officinalis, can significantly reduce the polarization of M1 macrophages and the expression of M1 macrophage-associated pro-inflammatory chemokines and cytokines in UC mice, which is beneficial for the treatment of UC (43). The Tongxie-Yaofang formula reduces M1 polarization of macrophages by inhibiting the activation of the NF-κB/NLRP3 signaling pathway and significantly increases M2 polarization, leading to a significant improvement effect on acute colitis (44). Dioscin, a traditional Chinese medicine compound isolated from Dioscorea, has powerful anti-inflammatory and immunomodulatory effects. In experiments using Dioscin to interfere with DSS-induced colitis in mice, it was found that Dioscin can improve colitis in mice, reduce the polarization of M1 macrophages, and significantly promote the polarization of M2 macrophages (45). The Huanglian-Houpo Decoction (HLHP) is a typical prescription for treating gastrointestinal diseases in ancient Chinese medicine. A study has shown that HL and HP (EHLHP) can regulate the polarization to M2 macrophages, reduce the level of inflammation, repair the intestinal mucosal barrier, and improve UC symptoms in experimental animals (46). Cayratia japonica (CJ) is a widely used folk medicine whose two main ingredients, rutin and quercetin, have been demonstrated to be effective in the treatment of inflammatory bowel disease (73). One study revealed that CJ can inhibit the TLR4/MAPK/NF-κB signaling pathway and macrophage M1 polarization. This promotes M2-like polarization, thereby reducing DSS-induced immunoinflammation in UC models and lessening colon damage (47). Berberine (BBR) is a common treatment for gastrointestinal disorders. It modulates inflammatory factors, decreasing the presence of M1 macrophages, and enhances STAT6 phosphorylation by activating downstream elements of the IL-4-STAT6 signaling pathway. This significantly promotes M2 polarization and effectively alleviates symptoms in UC mouse models (48). Didymin has the ability to convert the proinflammatory M1-like macrophage phenotype into an anti-inflammatory M2-like macrophage phenotype in an inflamed environment. However, it does not alter the polarization of M2-like macrophages (49). Wumeiwan (WMW) is commonly used to treat diarrhea resulting from colitis. A study has demonstrated that WMW inhibits intestinal inflammation and repairs damaged intestinal mucosa by suppressing the activation of p38MAPK, NF-κB, and STAT6 signaling pathways. It also inhibits M1 polarization, promotes M2 polarization, and regulates the levels of inflammatory factors (50). Cinobufacini, a compound with anti-inflammatory and anti-tumor properties, has demonstrated the ability to decrease M1 macrophage counts, significantly increase M2 macrophage numbers, and profoundly inhibit M1 polarization, thereby improving colitis (51). Sanguisorba officinalis L. (SOL), known for its potent anti-inflammatory effects, contains a component called Methyl gallate (MG) that exhibits efficient anti-UC efficacy. A study has revealed that MG can effectively prompt the polarization of macrophages from M1 to M2 and inhibit the release of inflammatory cytokines (52).

### 5.3 Restoration of the balance between T helper cells

Colitis is classified as an inflammatory bowel disease characterized by an imbalance in the immune response, involving both pro-inflammatory (Th1) and anti-inflammatory (Th2) cytokines (74). T helper cells are critical in facilitating adaptive immune responses and inflammatory reactions, including autoimmunity, asthma, and allergies, by reacting to specific pathogens or autoantigens. CD4^+^ T cells are divided into Th1 cells and Th2 cells based on their cytokine secretion profile. Under normal conditions, there is a dynamic balance between Th1 and Th2 in the body. However, when tissue inflammation occurs, this balance is disrupted, and an excess of Th1 and Th2 can contribute to the development of autoimmune diseases (75).

Buckwheat (F. esculentum) bee pollen extract (FBPE) is abundant in nutritional components, including luteolin, resveratrol, kaempferol, and other active ingredients. Research has demonstrated that it can regulate immune function, restore the Th1/Th2 balance, thereby alleviating clinical symptoms and tissue damage in colitis-induced mice. Furthermore, FBPE enhances intestinal epithelial barrier function and exhibits a protective effect on colitis (53). Platycodon grandiflorus polysaccharide (PGP), a main component of P. grandiflorus, has been shown to effectively reduce the levels of Th1 or Th17 related cytokines and transcription factors, while increase the levels of Th2 or Treg related counterparts, and treat UC by regulating the immune balance between Th1/Th2 and Th17/Treg cells (54). One study applied Periplaneta americana (P. americana) to acute colitis mice model, and the results showed that P. americana can regulate cytokines. The significantly elevated Th1/Th2 ratio in a mouse model of acute colitis was restored to normal, and DSS induced inflammation was suppressed through immunomodulatory effects (55).

### 5.4 Others

In cases of gastrointestinal inflammation, specific amino acids and metabolites are involved in the inflammatory process. The intervention of ginsenoside Rg1 (Rg1) can affect various metabolic pathways in the gut microbiota, including the regulation of valine, leucine, and vitamin B6 metabolism. The most notable impact is seen in the regulation of tryptophan metabolism. Rg1 has the ability to elevate the levels of tryptophan metabolites, which helps protect the intestinal barrier and decrease colon inflammation in mice with UC (56).

## 6 Chinese herbs regulate intestinal flora

The role of gut microbiota dysbiosis in the development and progression of various diseases has been increasingly supported by evidence (25). Given the similarities between IBD and ICIIC with other chronic intestinal inflammations, regulating the intestinal flora becomes crucial in the treatment of intestinal disorders. TCM follows the principle of “treating different diseases using the same method,” which is a fundamental concept in TCM theory. When multiple diseases share a common underlying cause, TCM practitioners often prescribe similar or identical treatments. Network pharmacology research has also identified shared pathogenic mechanisms across different diseases, allowing TCM to address multiple conditions by targeting these core factors (25). Numerous studies have shown that herbal medicine’s efficacy is closely related to improving gut microbiota.

Currently, studies have shown a relevant connection between the intestinal flora and irAEs. Manipulating the gut microbiota has demonstrated significant and rapid improvement in irAEs associated with ICIs, as observed in both preclinical and clinical trials. A case series reported successful treatment of ICIIC through fecal microbiota transplantation, resulting in the reconstruction of the intestinal microbiome and an increased proportion of regulatory T cells in the colon mucosa, highlighting the potential of modulating the intestinal microbiome to mitigate ICIIC (20). The study showed that colitis in ICIIC patients predominated with *Clostridia* and *Escherichia*, the latter of which is associated with an intestinal disorder. After treatment, the increase of *Akkermansia*, *Bacteroidia*, *Blautia* and *Bifidobacterium* was associated with the improvement of colitis (20). *Lactobacillus reuteri* has demonstrated alleviation of ICIIC by downregulating group 3 innate lymphocytes (ILC3s) (76). Wang et al. (77) proved that the combinations of anti-CTLA-4 and anti-PD-1 antibody treatments induced colitis, in the mouse model, fecal microbial sequencing showed that the abundance of *Lactobacillus* in the intestinal flora of mice with immune checkpoint associated colitis was significantly reduced. Many studies have shown that gut microbiota is also involved in Th17/Treg balance (78, 79), such as *Lactobacillus* and *Bifidobacterium* are involved in Treg development and T cell response (80). In addition, a prospective study revealed that ipilimumab treatment did not change the microbiome composition, while the occurrence of ICIIC was associated with a decrease in microbial diversity, such as several genera in *Firmicutes* were significantly reduced (11). Improving intestinal flora richness to improve intestinal flora disturbance is one of the functions of TCM. Rhine up-regulates *Bacteroidetes*, especially *Rikenellaceae*, a kind of *Bacteroidetes*, which can enhance the barrier function of intestinal epithelial cells, play a beneficial role in anti-inflammation and UC, and is negatively correlated with pro-inflammatory factors, such as IL-1β, IL-6 and TNF-α. It can effectively inhibit the production of proinflammatory cytokines (57). Another study showed that Rhine can also increase lactobacillus levels, protect the intestinal barrier, and reduce colitis (58). Berberine (BBR), an isoquinoline alkaloid derived from various Chinese herbal medicines, has been extensively utilized in the treatment of dysentery and colitis. In a study, it was found that the gut microbiota can metabolize BBR into Oxyberberine (OBB) through an oxidation reaction. Oxyberberine (OBB) possesses a spectrum of pharmacological benefits, encompassing anti-inflammatory, anti-fungal, anti-tumor, and anti-arrhythmic properties, with OBB outperforming BBR in terms of anti-inflammatory, anti-fungal, and anti-arrhythmic efficacy. This study showed that both OBB and BBR can increase the abundance of Bacteroides, and OBB has superior anti-colitis activities, including improving the intestinal mucosal barrier, inhibiting colitis tissue damage, and reducing pro-inflammatory factors such as IL-6, IL-1β, IL-17, TNF-α and IFN-γ (59). Coptis chinensis Franch (referred to as Huanglian in Chinese, HL) and Magnoliae officinalis (known as Houpo, HP) have been traditionally used in folk medicine for hundreds of years to treat gastrointestinal disorders, including ulcers and inflammation. A study demonstrated the therapeutic effects of HL and HP on 2,4, 6-trinitrobenzene-sulfonic acid-induced UC rats and their effects on intestinal flora in UC rats. The results showed that HL+HP supplementation augmented the abundance of beneficial bacteria such as Akkermansia and Blautia, significantly reduced the harmful flora, improved colitis, and demonstrated the ability to suppress inflammatory responses (60). Gegen-Qinlian decoction (GQD) stands as a classic formulation in TCM, comprising four medicinal herbs: Puerariae Lobatae Radix, Scutellariae Radix, Coptidis Rhizoma, and Glycyrrhizae Radix et Rhizoma Praeparata cum Melle. GQD demonstrates efficacy in treating inflammatory intestinal diseases, encompassing diarrhea, ulcerative colitis, and intestinal adverse reactions induced by chemotherapy drugs. Studies have revealed that GQD treatment enhances the diversity of gut bacterial species and reshapes the composition of gut microbial communities. Specifically, GQD treatment increased the relative abundance of gut bacteria, including Akkermansia, Bacteroides and others (61). Another study showed that GQD treatment of colitis also significantly reduced TNF-α, IL-6, IL-1β, and IL-4 (62). Geng, known as the “king” of TCM, exhibits notable therapeutic effects on various diseases. Treatment with ginseng has been shown to increase the abundance of beneficial probiotics such as Bifidobacterium, Bacteroides, and Akkermansia, and improve colitis and diarrhea effectively by increasing Bacteroidetes and Lactobacillus. Furthermore, certain bacteria, including *Bacteroides*, *Eubacterium*, and *Bifidobacterium*, have been found to transform ginsenosides, improving the absorption rate of ginseng to improve colitis (63). Pingkui enema, a herbal compound used to treat intestinal diseases, has been shown to have a significant therapeutic effect on TNBS-induced UC by increasing the content of Bifidobacterium and adhesin receptors of bifidobacterium, reducing the concentration of IL-8 and TNF-α, and increasing the concentration of IL-13 (64). Rabdosia serra (R. serra) can increase the abundance of Bacteroides, Lactobacillus and other beneficial bacteria, regulate immune factors, and restore the balance of Treg/Th17 to treat colitis (65).

## 7 Exploration on the treatment approaches for ICIIC from a TCM standpoint

In traditional Chinese medicine theory, long-term improper diet and emotional stimulation can impact visceral function and impair the body’s healthy qi. A deficiency in healthy qi can weaken the body’s ability to resist external pathogens and clear abnormal cells. Additionally, prolonged visceral dysfunction, abnormal transport, phlegm stagnation, blood stasis, and the accumulation of turbid phlegm can contribute to cancer toxin buildup, ultimately leading to tumor formation. Therefore, the occurrence of tumors is believed to result from the struggle between healthy and pathogenic qi within the human body.

**Figure 2:**
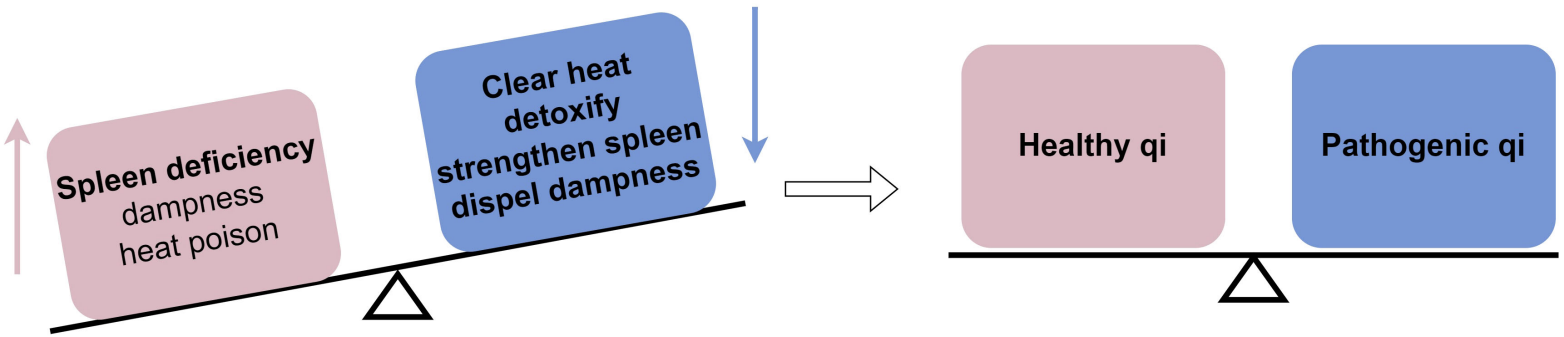
Mechanism by which TCM affects immune checkpoint inhibitor-induced colitis from a TCM standpoint: TCM helps the body achieve a balance between healthy qi and pathogenic qi by eliminating pathogenic factors.

ICIs are a novel category of immunotherapy drugs used for anti-tumor treatment by activating the immune system to eliminate cancer cells through immune checkpoint blockade. Although PD-1/PD-L1 immunosuppressants have demonstrated significant effectiveness in this regard, their mechanism of action can disrupt the balance of the body’s immune environment, leading to irAEs. Colitis, a common side effect associated with immune checkpoint inhibitors, is categorized in TCM as “diarrhea” or “dysentery,” with diarrhea being the primary clinical symptom. According to TCM, the key pathogenesis of this condition is spleen deficiency with dampness. Spleen deficiency is characterized by digestive issues and imbalanced enteric microflora. Dampness accumulation transforms into heat, leading to the build-up of heat toxicity internally. Clinical indicators include sticky, sluggish stools, and tenesmus. Elevated levels of pro-inflammatory cytokines can result in heightened systemic inflammatory responses, increased vascular permeability, and damage to visceral tissues. Therefore, the guiding principle for treating this condition involves fortifying the body’s resistance to eradicate the pathogenic factors. The treatment method focuses on clearing heat and detoxification, as well as strengthening the spleen and dispelling dampness (**Figure 2**).

In TCM, Codonopsis is known for its spleen-tonifying properties. Codonopsis polysaccharide, a compound derived from the herb Codonopsis, has demonstrated immunomodulatory effects, particularly in maintaining immune balance. Thus, it may help address the immune imbalance caused by ICIs (81). Gegen Qinlian decoction (GGQLD) and Huanglian Jiedu decoction (HLJDD) are renowned for their heat-clearing properties and have been found to possess significant anti-inflammatory effects. This makes them suitable for the treatment of acute or chronic inflammatory diseases. Huanglian Decoction (HLD) has been shown to alleviate the symptoms of DSS-induced colitis, reduce histological damage, downregulate the level of pro-inflammatory cytokines, and improve dysfunctional intestinal flora (82). Baitouweng decoction and Huangqin decoction (HQD) are known for their ability to “clear heat and stop diarrhea” and are commonly utilized in the treatment of damp-heat type ulcerative colitis. HQD has been demonstrated to reduce pro-inflammatory factors, regulate intestinal flora, effectively alleviate symptoms in mice with ulcerative colitis, and improve their mental state (83). Shenling Baizhu powder has been demonstrated to enhance human immune function, effectively alleviating diarrhea symptoms, regulating immune factors, and improving intestinal flora (84, 85). Shaoyao decoction (SYD) is a TCM prescription with a history dating back to the Jin-Yuan Dynasty. It is commonly employed for the treatment of various inflammatory bowel diseases and intestinal dampness-heat syndrome. Research has indicated that SYD can significantly inhibit the production of pro-inflammatory cytokines and chemokines, especially IL-1β, IL-6, and IFN-γ. Moreover, it has been demonstrated to markedly reduce intestinal mucosal damage in DSS-induced UC mouse models and protect the intestinal barrier (86). Sanhuangshu’ai decoction (SH) is a combination of four commonly used Chinese herbal medicines: Coptidis Rhizoma, Scutellariae Radix, Phellodendri Chinensis Cortex, and Artemisiae Argyi Folium. Research has indicated that this decoction can effectively improve colitis in mice induced by DSS (87).

## 8 Conclusion

Tumors are severe diseases that pose a significant threat to human health and diminish overall quality of life. Although tumor immunotherapy offers new hope for countless cancer patients, frequent adverse reactions and serious side effects often expose patients to additional risks. Traditional Chinese medicine has been widely used in clinical practice in China, particularly for managing side effects caused by conventional cancer treatments. The principle of “same treatment for different diseases” in TCM suggests its wide applicability in treating various inflammatory intestinal diseases, an effectiveness that has been supported by experts and scholars. Exploring the potential of using TCM for immune checkpoint inhibitor-induced colitis could offer a novel therapeutic approach and method for clinical treatment, bringing much-needed relief to many patients and significantly improving their quality of life. In future, there is promise in discovering and implementing additional TCM approaches that can prevent and alleviate immune-related adverse events in clinical settings. However, TCM research on irAEs is still in its early stages, with limited available literature. A wealth of clinical and practical research evidence is needed to establish the efficacy of TCM and provide better clinical guidance for the prevention and treatment of irAEs using TCM.

## Author contributions

JW: Writing – original draft. ZG: Writing – original draft. MS: Writing – review & editing. QX: Supervision, Writing – review & editing. HX: Supervision, Writing – review & editing.
